# Estimating genomic breeding values and detecting QTL using univariate and bivariate models

**DOI:** 10.1186/1753-6561-5-S3-S5

**Published:** 2011-05-27

**Authors:** Mario PL Calus, Han A Mulder, Roel F Veerkamp

**Affiliations:** 1 Animal Breeding and Genomics Centre, Wageningen UR Livestock Research, Lelystad, Netherlands

## Abstract

**Background:**

Genomic selection is particularly beneficial for difficult or expensive to measure traits. Since multi-trait selection is an important tool to deal with such cases, an important question is what the added value is of multi-trait genomic selection.

**Methods:**

The simulated dataset, including a quantitative and binary trait, was analyzed with four univariate and bivariate linear models to predict breeding values for juvenile animals. Two models estimated variance components with REML using a numerator (A), or SNP based relationship matrix (G). Two SNP based Bayesian models included one (BayesA) or two distributions (BayesC) for estimated SNP effects. The bivariate BayesC model sampled QTL probabilities for each SNP conditional on both traits. Genotypes were permuted 2,000 times against phenotypes and pedigree, to obtain significance thresholds for posterior QTL probabilities. Genotypes were permuted rather than phenotypes, to retain relationships between pedigree and phenotypes, such that polygenic effects could still be estimated.

**Results:**

Correlations between estimated breeding values (EBV) of different SNP based models, for juvenile animals, were greater than 0.93 (0.87) for the quantitative (binary) trait. Estimated genetic correlation was 0.71 (0.66) for model G (A). Accuracies of breeding values of SNP based models were for both traits highest for BayesC and lowest for G. Accuracies of breeding values of bivariate models were up to 0.08 higher than for univariate models.

The bivariate BayesC model detected 14 out of 32 QTL for the quantitative trait, and 8 out of 22 for the binary trait.

**Conclusions:**

Accuracy of EBV clearly improved for both traits using bivariate compared to univariate models. BayesC achieved highest accuracies of EBV and was also one of the methods that found most QTL. Permuting genotypes against phenotypes and pedigree in BayesC provided an effective way to derive significance thresholds for posterior QTL probabilities.

## Background

Genomic selection is particularly beneficial for difficult or expensive to measure traits [1]. One strategy to partly tackle these issues in breeding schemes previously, without using genotypic information, was multi-trait selection [e.g. 2]. An important question is therefore what the added value is of multi-trait genomic selection. VanRaden and Sullivan [3] showed some benefit using this approach in international dairy cattle evaluations. There are, however, no other reports so far on applications of multi-trait genomic selection. The objective of this study was to present methods to apply multi-trait genomic breeding value prediction, and to evaluate their performance and impact on accuracy of prediction compared to single trait applications. In addition, the ability of one model to detect QTL was investigated.

## Methods

### Estimation of breeding values

Simulated data of the 14^th^ QTL-MAS workshop was analyzed with univariate and bivariate applications of four different models to predict breeding values for juvenile animals without phenotypes. A linear model was assumed for both the quantitative and binary trait. Using a linear model for binary traits is expected to give breeding values that are highly related to those obtained from a threshold model, when trait incidence is moderate [e.g. 4], which is the case here with a value of 0.30. The first two models used ASREML to estimate variance components:

*y_ij_* = *µ_j_* + *animal_ij_* + *e_ij_*

where *y_ij_* is the phenotypic record of animal *i*, *µ_j_* is the overall mean for trait *j*, *animal_ij_* is the random polygenic effect of animal *i* for trait *j*, and *e_ij_* is a random residual for animal *i*. Model A used a numerator relationship matrix for polygenic effects, while model G used a SNP based genomic relationship matrix. For G, matrix **G** was calculated as [5]:

,

where **Z** contained marker genotypes for all animals across loci, being -1 and 1 for either homozygote and 0 for the heterozygote genotype, corrected for allele frequency per locus in the current population.

The third and fourth model were based on Gibbs sampling and included SNP effects, next to the pedigree based relationship matrix:

where *SNP_ijkl_* is a random effect for allele *l* on trait *j* at locus *k* of animal *i*. The difference between those two models is that 1 (BayesA) or 2 (BayesC) distributions for SNP effects are considered, respectively.

SNP effects, denoted as *SNP_ijkl_*, were estimated in BayesA and BayesC as *q_ijkl_*×*v_.k_*[6], where *q_ijkl_* is the effect size of allele *l* at locus *k* and *v_.jk_* is the direction vector for locus *k* that scales the effect at locus *k* for trait *j*. In the original implementation [6], variance of the direction vector *v_jk_*, denoted as **V**, is sampled for each trait *j* separately, without considering covariances between traits across loci. Here, both in BayesA and BayesC, in **V** covariances between traits across loci are considered.

### QTL mapping

BayesC, also known as Bayesian stochastic search variable selection (BSSVS) [7], involved sampling presence of a QTL at each SNP position from a Bernoulli distribution with probability equal to , where P(**v_j_** | **0**, **V**) is the probability of sampling **v_j_** from N(**0**, **V**), and Pr_j_ is the prior probability of presence of a QTL at SNP position *j*. Pr_j_ was calculated per locus as 50 divided by the total number of SNPs, reflecting that 50 QTL were expected. Posterior QTL probabilities were calculated as proportions of cycles after burn-in that a locus was placed in the distribution with large effects and therefore was sampled from N(**0**, **V**). For more details on prior distributions and fully conditional distributions, see Meuwissen and Goddard [6].

To obtain significance thresholds for posterior QTL probabilities for the bivariate BayesC model, genotypes were permuted 2,000 times against phenotypes and pedigree.

## Results

### Variance components

Estimated variance components obtained from bivariate models A and G were used to calculate heritabilities and genetic correlations (Table 1). Estimated heritabilities and genetic correlations were not significantly different between bivariate models A and G. Standard errors of heritabilities and genetic correlations were lower for G than for A.

**Table 1:** Estimated heritabilities and genetic correlations.

	h^2^					
			
Model	Quantitative	s.e.	Binary	s.e.	r_g_	s.e.
A	0.53	0.06	0.22	0.04	0.66	0.09
G	0.46	0.03	0.29	0.03	0.71	0.06

Estimated heritabilities and genetic correlations (r_g_), and standard errors (s.e.), obtained with models with an additive genetic (A) or SNP based relationship matrix (G).

### Breeding values

Correlations were calculated among EBVs of all models for juvenile animals (Table 2). EBV of model A had correlations with SNP based models of ~0.6 for both traits. EBV were highly correlated between G and BayesA and BayesA and BayesC, but the correlation dropped to 0.94-0.95 when comparing G and BayesC. EBV of univariate and bivariate models had correlations of ≥0.98 for the quantitative trait, and ≥0.93 for the binary trait.

**Table 2:** Correlations between predicted breeding values of juvenile animals.

		Univariate				Bivariate			
		
		A	G	BayesA	BayesC	A	G	BayesA	BayesC
		
	A		0.60	0.67	0.63	0.99	0.62	0.61	0.58
Uni	G	0.60		0.98	0.94	0.60	0.99	0.99	0.94
	BayesA	0.62	1.00		0.98	0.66	0.98	0.99	0.96
	BayesC	0.56	0.95	0.96		0.63	0.94	0.96	0.98
	A	0.93	0.62	0.64	0.60		0.63	0.61	0.58
Biv	G	0.60	0.95	0.95	0.94	0.64		0.99	0.95
	BayesA	0.58	0.94	0.95	0.96	0.63	0.99		0.98
	BayesC	0.50	0.88	0.88	0.95	0.57	0.94	0.96	

Correlations between breeding values predicted using univariate (Uni) and bivariate (Biv) models with an additive genetic (A) or SNP based relationship matrix (G), and a Bayesian model with one (BayesA) or two distributions (BayesC) for SNP effects. Correlations above (below) the diagonal are for the quantitative (binary) trait.

Accuracies increased markedly going from A to G, and less so going from G to BayesA and from BayesA to BayesC (Table 3). Accuracies were always higher for bivariate than for univariate models. The relatively small increase in accuracy for bivariate compared to univariate models, especially for the quantitative trait, indicates that added power in the bivariate model was limited for this trait. Coefficients of regressing true breeding values on estimated breeding values were <1.0 for all models, indicating that variance of EBV was generally overestimated (Table 3). This overestimation was smaller for SNP based models, compared to model A.

**Table 3:** Accuracies and regressions of true on estimated breeding values for juvenile animals.

	Accuracy				Regression coefficient			
	
	Quantitative trait		Binary trait		Quantitative trait		Binary trait	
Model	Uni.	Biv.	Uni.	Biv.	Uni.	Biv.	Uni.	Biv.
A	0.39	0.39	0.47	0.52	0.84	0.84	0.71	0.75
G	0.61	0.62	0.72	0.79	0.96	0.96	0.83	0.88
BayesA	0.63	0.64	0.73	0.81	0.96	0.96	0.84	0.91
BayesC	0.66	0.67	0.79	0.85	0.93	0.93	0.91	0.95

Correlations between true and estimated breeding values, and coefficients of regressions of true on estimated breeding values, predicted using univariate (Uni.) and bivariate (Biv.) models with an additive genetic (A) or SNP based relationship matrix (G), and a Bayesian model with one (BayesA) or two distributions (BayesC) for SNP effects

### QTL detection

Detection of QTL was considered for univariate and bivariate BayesC models, while significance thresholds were only derived for the bivariate BayesC model. Therefore, only detected QTL from the bivariate BayesC model were used in the comparison of QTL detection methods.

For the quantitative trait 14 out of 32 QTL were detected, while for the binary trait 8 out of 22 were detected [8]. SNP that were declared significant together explained 35.0% and 22.6% of the genetic variance of the quantitative and binary trait, respectively. Polygenic effects explained only 4.3 and 1.1% of the genetic variance. This indicates that most of the genetic variance (i.e. 60.7 and 76.2% for the quantitative and binary trait, respectively) in the bivariate BayesC model was explained by effects of SNP that where not declared significant.

**Figure 1:**
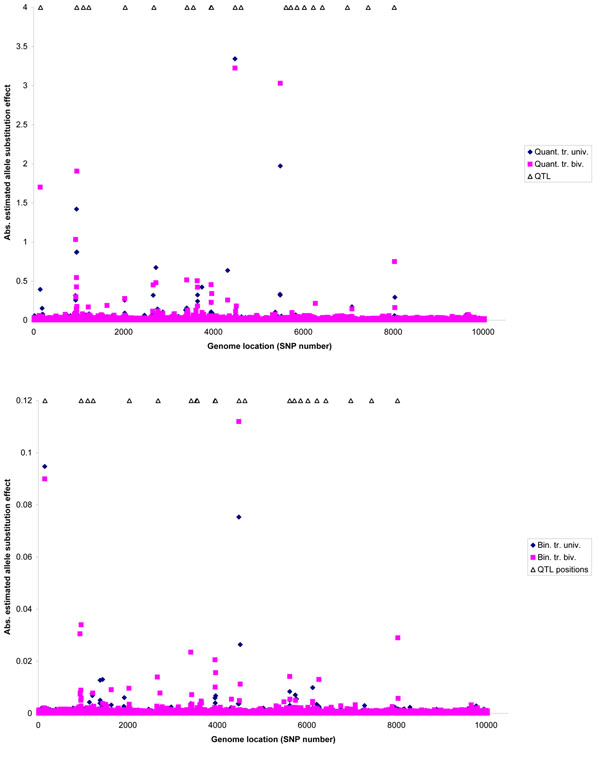
**Absolute allele substitution effects across the genome for the quantitative** (A) and binary trait (B), estimated using univariate and bivariate BayesC models.

Absolute allele substitution effects for both traits estimated with univariate and bivariate BayesC models were plotted across the genome, together with positions of all additive QTL (Figure 1). Generally, allele substitution effects from the bivariate model were higher than from the univariate model. Since most additive QTL were pleiotropic, the bivariate model indeed seemed able to use information from both traits to estimate SNP effects per trait more accurately.

## Discussion

This study aimed to present methods to apply multi-trait genomic breeding value prediction, to evaluate impact on accuracy of prediction compared to single trait genomic breeding value prediction, and to detect QTL with one of the models. Results clearly indicated that accuracy of EBV increased when model complexity increased to allow better modeling of the genetic architecture. First, accuracy increased going from model A, to SNP based models with increasing flexibility to model SNP effects (in the order: G, BayesA, BayesC). Second, accuracy of EBV for both traits increased more for all SNP based models when using bivariate instead of univariate applications, compared to model A. This confirms results of a simulation study for dairy cattle showing that model G yields higher accuracies when using data of multiple countries compared to one country [3]. Third, considering that few QTL had relatively large effects, it was expected that the model best able to give more weight to loci with large effect – BayesC – fits the data best. Results are fully in agreement with this expectation. This suggests that SNP based models were better able to capture pleiotropic effects of QTL.

The model that achieved highest EBV accuracy, i.e. BayesC, was also one of the presented models that detected most QTL. The model that is best able to detect the position of QTL, however, is not always the model that is best able to predict total genetic merit of animals [9]. Permuting genotypes against phenotypes and pedigree in model BayesC provided an effective way to derive significance thresholds for posterior QTL probabilities. Note that SNP genotypes after the permutation no longer followed Mendelian inheritance. Lack of Mendelian inheritance probably results in fewer associations, since SNPs are less likely to capture pedigree effects, and therefore a lower threshold. In the present data, however, polygenic effects only captured a very small fraction of the variance, indicating that the applied permutation strategy will have had a minor impact on significance thresholds.

## Conclusions

The EBV accuracy clearly improved for both traits for all bivariate models compared to their univariate counterparts. BayesC achieved highest EBV accuracies and was also one of the methods presented at the workshop that found most QTL.

## Competing interests

The authors declare no competing interests.

## Authors' contributions

MPLC developed software for the Bayesian models, carried out analyses and drafted the manuscript. HAM and RFV helped to interpret results and write the manuscript. All authors read and approved the final manuscript.
